# A Personalized Approach to Adhesion Prevention in Single-Port Access Laparoscopic Surgery: A Randomized Prospective Study Evaluating the Efficacy of Adhesion Barriers and Patient-Specific Risk Factors

**DOI:** 10.3390/jpm15020068

**Published:** 2025-02-12

**Authors:** Seongyun Lim, Joseph Noh, Junhyeong Seo, Youngeun Chung, Taejoong Kim

**Affiliations:** 1 Division of Gynecologic Oncology, Department of Obstetrics and Gynecology, Samsung Medical Center, Sungkyunkwan University School of Medicine, Seoul 06351, Republic of Korea; sy4577.lim@samsung.com (S.L.); junhyeong.seo@samsung.com (J.S.); y9242.chung@samsung.com (Y.C.); 2 Division of Gynecologic Oncology, Department of Obstetrics and Gynecology, Seoul National University Bundang Hospital, Seongnam 13620, Republic of Korea

**Keywords:** single-port laparoscopy, single-incision laparoscopic surgery (SILS), postoperative adhesion, postoperative complication, minimally invasive laparoscopic surgery

## Abstract

**Background/Objectives**: Single-port access (SPA) laparoscopic surgery has gained popularity due to its cosmetic benefits and reduced postoperative pain. However, concerns persist regarding the increased risk of adhesions due to the larger umbilical incision. This study aims to contribute to personalized medicine by evaluating the effectiveness of applying an anti-adhesive agent (Guardix SG^®^, HanmiPharmaceutical Co., Ltd., Seoul, Korea) at the umbilical incision and identifying patient-specific risk factors for adhesion formation in SPA laparoscopic surgeries. **Methods**: In this randomized prospective study, 55 female patients with benign gynecological conditions were enrolled. Participants were randomly assigned to either an intervention group, which received the anti-adhesive agent at both the surgical and umbilical sites, or a control group, which received it only at the surgical site. Participants returned for outpatient visits 1–3 months post-surgery to assess incision site complications, including adhesions. **Results**: The overall adhesion rate was 10.9%, with 13.3% in the control group and 8% in the intervention group, though the difference was not statistically significant (*p* = 0.678). Infection rates were 6.7% in the control group and 4% in the intervention group; however, there was no significant difference in complications. Logistic regression identified pre-existing adhesions as a significant risk factor (*p* = 0.0379; OR = 6.909). **Conclusions**: Although the adhesion barrier showed a trend toward reducing umbilical adhesions, the difference was not statistically significant. The application of the adhesion barrier did not influence incision site complications, confirming its safety. Our findings highlight the need for personalized approaches to adhesion prevention, considering individual patient characteristics and risk factors. Further larger studies are necessary to explore adhesion prevention in a more personalized manner for individual patients in this context.

## 1. Introduction

In recent years, the paradigm of surgery has shifted from traditional open surgery to minimally invasive techniques such as laparoscopic and robotic surgeries. Among these, single-port access (SPA) laparoscopic surgery, involving the insertion of a camera and surgical instruments through a single incision, has become popular due to its cosmetic benefits and reduced postoperative pain, which is related to the patient’s quality of life. Single-port laparoscopic surgery is widely and safely employed in gynecology for procedures such as ovarian cystectomy, salpingo-oophorectomy, myomectomy, and hysterectomy [1,2]. 

However, concerns have been raised about the risk of adhesions associated with the relatively larger incision at the umbilicus compared to conventional laparoscopic surgery. One study reported that doubling the port size doubles the incidence of adhesions [3]. While conventional laparoscopy involves incisions of 5 to 12 mm, SPA laparoscopy necessitates an umbilical incision of 2.0–3.0 cm, potentially increasing adhesion risk by two to six times.

Postoperative adhesions are associated with numerous complications such as small bowel obstruction, infertility, and chronic pelvic pain [4,5]. In the era of personalized medicine, understanding individual patient factors that contribute to postoperative adhesion formation is crucial. Factors such as incision type and underlying conditions have been the primary focus of research on adhesion formation [6,7,8]. Recent studies have focused on the genetic factors that may predispose individuals to postoperative adhesion formation [9,10,11].

To prevent such adhesions, anti-adhesive agents that act as physical barriers to reduce adhesion formation between adjacent tissues are commonly employed in clinical settings. The efficacy of anti-adhesive agents in preventing surgical site adhesions in open surgery has been well-documented in numerous studies [12]. However, research on postoperative adhesions in laparoscopic surgery, particularly single-port laparoscopy, remains limited.

In a previous single-arm study, the author confirmed the absence of postoperative adhesions, wound dehiscence, or infections following the application of an adhesion barrier [13]. This randomized prospective study aims to evaluate the incidence of incisional adhesions in gynecological patients undergoing SPA laparoscopic or robotic surgery. Additionally, it aims to contribute to personalized medicine by identifying patient-specific risk factors that may predispose individuals to adhesion formation after SPA laparoscopic surgery. Furthermore, the study assesses the incidence of incisional site complications such as dehiscence and infection following SPA laparoscopic surgery

## 2. Materials and Methods

### 2.1. Patient Group

The patient group comprised female individuals aged 19 to 65 with benign gynecological conditions, including ovarian cysts, fibroids, and adenomyosis. The exclusion criteria were as follows: (1) a history of abdominal or pelvic surgery, (2) pre-existing conditions causing abdominal or pelvic adhesions, (3) evidence of abdominal or pelvic adhesions in preoperative imaging, (4) current pregnancy or lactation, (5) a history of severe drug allergies, (6) an inability to comprehend the study due to physical or mental impairments, (7) participation in another clinical trial within the previous 30 days, (8) any other medical judgment deeming participation inappropriate (e.g., non-cooperation), (9) an inability to attend the hospital for ultrasound within three months post-surgery.

Patients who voluntarily signed the informed consent form were screened to assess their eligibility. Suitable participants were randomly assigned to either the intervention or control group using a randomization table. The patients were blinded to their treatment allocation, and the evaluators were not involved in the application of the investigational medical device, maintaining a double-blind design.

### 2.2. Surgical Method and Application of Adhesion Barrier

Both the intervention and control groups underwent a surgical procedure performed by the same surgeon (TJ Kim) using a single-port platform inserted at the umbilicus for either conventional laparoscopy or Da Vinci Xi single-site robotic surgery, based on patient preference. A 2.0–3.0 cm umbilical incision was made using the open Hasson technique.

Postoperatively, Guardix SG^®^ (HanmiPharmaceutical Co., Ltd., Seoul, Korea), an adhesion barrier, was applied to the pelvic surgical site in both groups. In the intervention group, Guardix SG^®^ was also applied beneath the umbilical port before suturing the fascia and peritoneum. In the control group, Guardix SG^®^ was applied only to the pelvic site without additional placement beneath the umbilical port. Guardix SG^®^ was used under the peritoneum around the umbilical opening with a right-angled applicator, designed for use through the umbilical incision. The amount used at the umbilical site was 6 g, which is sufficient to cover the entire area around the opening. Guardix SG^®^ is a temperature-sensitive anti-adhesive agent that prevents postoperative adhesions. To ensure that Guardix SG^®^ gelifies adequately at body temperature, sufficient time was allowed before starting the suturing process. The suturing technique and postoperative patient care were identical in both groups.

### 2.3. Adhesion Assessment Method (Visceral Sliding Technique)

**Figure 1:**
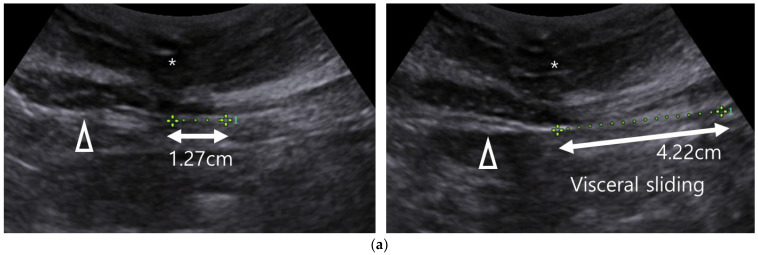

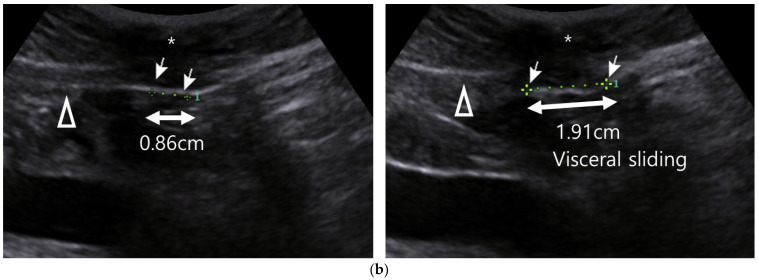
Visceral sliding technique via trans-abdominal ultrasound. Visceral sliding distances during tidal and maximal respiration are relatively shorter in cases with adhesions. (**a**) Visceral sliding during tidal and maximal respiration in a patient without adhesions; (**b**) visceral sliding during tidal and maximal respiration in a patient with adhesions. (*) Rectus abdominis muscle, (Δ) visceral peritoneum; Supplementary Video S1.

Participants from both groups returned for an abdominal ultrasound 1–3 months after surgery. The visceral sliding technique is effective in assessing periumbilical adhesions. During respiration, abdominal organs move vertically or horizontally with diaphragm movement; adhesions impede this movement [14]. The criteria for determining adhesion using the visceral sliding technique were based on a meta-analysis study [15]. According to the study, the sensitivity of the visceral sliding technique was retrospectively confirmed to be 91.1% (80.5–96.2%) and the specificity was 93.2% (83.3–97.4%). A vertical organ movement distance of ≥1.0 cm was considered normal. Movement <1.0 cm, either during tidal or maximal respiration, was classified as restricted viscera slide, indicating adhesions (Figure 1). If patients could not follow the instructions, manual ballottement with an ultrasound probe was used to assess organ mobility. Two independent gynecologic oncologists, unaware of treatment assignment, conducted assessments using random screening numbers.

### 2.4. Variables

Age, height, and weight were recorded on admission day prior to surgery to calculate BMI. Blood tests were performed within one month before surgery and on the day following surgery to record hemoglobin differences. ASA scores for anesthesia and comorbidities such as hypertension, diabetes, and dyslipidemia were obtained from electronic medical records. The surgical diagnosis and method, the operation duration, pre-existing adhesions, and the amount of blood loss were documented using operation records. Postoperative adhesions were evaluated using the visceral sliding technique within three months after surgery; incisional site complications such as infections or dehiscence were also monitored.

Incisional site infection was defined as the presence of clinical signs of inflammation at the surgical site, such as redness, discharge, or tenderness, requiring antibiotic therapy. Superficial dehiscence of the incisional site was defined as separation at the subcutaneous level, with an intact fascia level, necessitating further suturing. Deep dehiscence was defined as separation extending to the fascia level. Incisional hernia was defined as the protrusion of an organ or other tissue through the wound.

### 2.5. Statistical Analysis

Statistical analyses were performed using R 4.4.1 (Vienna, Austria; http://www.r-project.org/, accessed on 26 December 2024). Continuous variables such as age and BMI are presented as means ± standard deviations. Dichotomous variables were analyzed using Fisher’s exact test. *p*-values less than 0.05 were deemed significant.

## 3. Results

**Figure 2:**
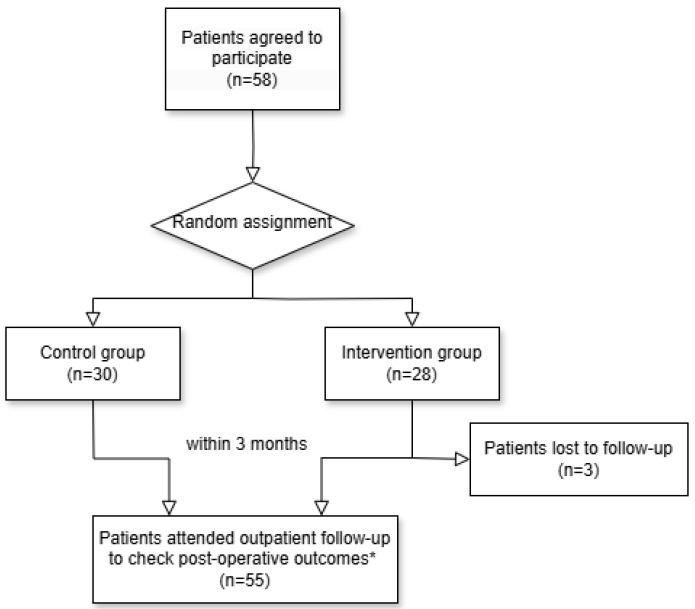
Study flow chart. * Postoperative outcomes include postoperative adhesion, surgical site infection, and dehiscence.

From 21 June 2022 to 8 February 2023, 58 patients consented to participate in this study. The intervention group received an adhesion barrier application beneath the umbilical surgical site, while the control group did not. In total, 58 patients were randomized, with 30 in the control group and 28 in the intervention group. Of the latter, three patients dropped out due to non-attendance within three months post-surgery, leaving 25 in the intervention group. Consequently, analyses were conducted for 55 patients (Figure 2). The timing of ultrasound follow-ups for patients was a median of 86 days (minimum of 59 days to maximum of 101 days).

Table 1 depicts the baseline characteristics of the patients. The average age was 38.9 years; the control group averaged 39 years, and the intervention group 38.7 years. The mean BMI was 23.1, with figures of 24.0 for the control group and 22.1 for the intervention group. Fourteen patients (25.5%) had comorbidities such as hypertension, diabetes, or hyperlipidemia, with eight in the control group and six in the intervention group.

Of the 55 surgeries, 32 (58%) were robotic and 23 (42%) were laparoscopic. There were 22 cases (40%) of adnexectomy procedures such as ovarian cystectomy or salpingectomy, and 16 cases (29%) of myomectomy. Hysterectomies, which often included adnexal procedures like salpingectomy, accounting for 14 cases (25.5%). There were two cases (3.6%) that combined myomectomy with adnexal surgery, and one case (1.8%) of adhesiolysis alone. Fifteen patients (27.3%) were diagnosed with endometriosis either preoperatively or intraoperatively, with ten in the control group and five in the intervention group.

The average duration of surgeries was 102.8 min, with 114.8 min for the intervention group and 92.8 min for the control group. The average blood loss was recorded at 158 mL, with 192.8 mL for the intervention group and 129 mL for the control group.

Despite the exclusion of patients with previous abdominal surgeries or adhesion-related histories from the study criteria, intra-abdominal adhesions were identified in 15 cases (27.3%). The severity of adhesions varied, but in this study, patients requiring adhesiolysis during surgery were classified as having intraoperative adhesions. There was one case (1.8%) in the intervention group that required a transfusion.

Table 2 summarizes the organ movement distances measured using the visceral sliding technique and the postoperative complications. The overall umbilical adhesion rate was 10.9%, with rates of 13.3% in the control group and 8% in the intervention group, indicating a relatively lower rate in the intervention group; however, Fisher’s exact test revealed no statistical significance between groups (*p*-value = 0.678). The postoperative wound infection rates were 6.7% for the control group and 4% for the intervention group. The incisional dehiscence rates were 3.3% in the control group and 0% in the intervention group, with no statistically significant differences in incision site complications between groups (*p*-value = 1). No incisional hernias were observed. None of the patients were re-admitted to the hospital within the follow-up period with symptoms suggestive of adhesions, such as bowel obstruction.

A sub-analysis was conducted on 40 patients without pre-existing pelvic adhesions to compare the control and intervention groups again (Table 3). The postoperative umbilical adhesion rate was 5%, with rates of 0% in the control group and 10% in the intervention group; however, no statistical significance was observed (*p*-value = 0.487). The wound infection rates of both groups were 5%, with dehiscence rates of 0% for both groups, indicating no statistically significant differences in incision site complications between groups (*p*-value = 1).

Logistic regression analysis was employed to identify risk factors for postoperative adhesion development, taking into account variables such as adhesion barrier application status, age, BMI, comorbidities, surgical modality, the presence of endometriosis, pre-existing adhesion, surgery duration, blood loss, and pre-and postoperative hemoglobin difference. Among these factors, only pre-existing adhesion was statistically significant as a risk factor (*p*-value = 0.0379; odds ratio = 6.909; 95% CI = [1.189–54.897]).

## 4. Discussion

SPA laparoscopic surgery is actively being introduced in various fields. Since there is only one incision site, it has led to increased satisfaction among gynecological patients due to its esthetic benefits, reduced pain, and faster recovery [16,17]. It is particularly useful in gynecologic surgery because the surgical site is relatively close to the umbilicus, and gynecological organs are symmetrically located and fixed within the pelvic cavity. Additionally, the use of a uterine manipulator allows for easier performance of SPA laparoscopic surgery. As it is being actively introduced, postoperative care and complications following SPA laparoscopic surgery are also important. While several studies have focused on postoperative complications such as infections and hernias [18], there has been little research focusing on adhesions at the incision site. This study was conducted as a prospective study, with emphasis on preventing such adhesions by applying an adhesion barrier at the umbilical incision site.

In this study, among the 55 patients, six cases of adhesions were observed, resulting in an incidence rate of 10.9%. We observed that the incidence of umbilical adhesion in patients who received an adhesion barrier was relatively lower at 8% compared to 13.3% in the control group, although this difference was not statistically significant. According to the Cochrane Database of Systematic Reviews, postoperative adhesions are observed in 1.6–51.0% of cases after general gynecologic laparoscopic surgeries [19].

The previous study conducted a single-arm study with 37 patients and reported an adhesion incidence rate of 0% [13]. However, it employed different criteria for detecting adhesions using the visceral sliding technique. While numerous studies discuss the efficacy of the visceral sliding technique, standardized criteria for detecting adhesions remain unestablished [15]. In that study, adhesions were defined as movement less than 1.0 cm during “both” tidal respiration and maximal inspiration. If the same criteria had been applied to our study population, only one patient in the control group out of 55 would have been diagnosed with adhesions. In this randomized controlled trial, we aimed to conduct a more comprehensive investigation of adhesions in SPA laparoscopic surgery, including risk factor analysis. We established different criteria, defining adhesions as movement less than 1.0 cm in either tidal respiration or maximal inspiration, thereby increasing sensitivity and decreasing specificity. This approach resulted in findings that differ from the 0% adhesion incidence rate reported in the prior study.

Nonetheless, this study has several significant implications. It is the first prospective randomized clinical trial to examine umbilical adhesions in SPA laparoscopic surgery. The adhesion rate at the SPA incision site in this study was 10.9% (8% in the intervention group and 13.3% in the control group), and among patients without previous adhesions, the rate was 5% (10% in the intervention group and 0% in the control group). Other studies have reported umbilical adhesion rates in conventional laparoscopic surgery ranging from 1.1% to 11.5% [20,21]. However, they used second-look laparoscopy for evaluation, while this study used ultrasound, potentially explaining some differences.

This study also presents data on complications related to the SPA incision site. The infection rates were 5.5% overall: 4% in the intervention group and 6.7% in the control group. The dehiscence rates were 1.8%, with 0% in the intervention group and 3.3% in the control group. The use of the adhesion barrier did not significantly affect the incidence of incision-related complications, indicating its safe application at the SPA incision site. However, further studies with a larger patient population are necessary to confirm these findings.

Furthermore, this study analyzed risk factors for adhesions at the SPA incision site. Known risk factors for adhesions in the literature include genetic variations in the interleukin-1 receptor antagonist, as well as clinical factors such as diabetes, endometriosis, obesity, and cancer [22]. In our analysis, factors like BMI, surgical method, and the presence of endometriosis were not significantly associated with adhesion formation, except for pre-existing adhesions. This suggests that pre-existing adhesions are a more critical factor, indicating that patients with a history of adhesions are more prone to developing additional adhesions, thus underscoring a strong genetic predisposition to adhesions and the need for careful monitoring in patients with prior adhesions to prevent postoperative umbilical adhesions. While the overall effectiveness of adhesion barriers was not statistically significant, our identification of pre-existing adhesions as a key risk factor suggests that patients with this characteristic may benefit more from targeted preventive measures. This personalized risk stratification could lead to more effective and efficient use of adhesion barriers, potentially improving patient outcomes and resource allocation. Future additional research could lead to the identification of high-risk groups for adhesion formation based on preoperative risk factors, potentially resulting in the development of tailored prevention strategies and postoperative care for these patient populations.

However, it remains unclear why the adhesion barrier did not significantly affect the umbilical incision site. Possible explanations include the small sample size, which may have been insufficient to detect significant effects. More extensive studies are likely required to ascertain these results.

Second, anatomically, adhesions tend to be less common in the umbilical region. The umbilicus is situated on the linea alba, which, in contrast to other sections of the abdominal wall, is devoid of muscle above the peritoneum and comprises only fascia, resulting in a relatively thin area due to minimal subcutaneous fat [23]. Furthermore, the umbilicus and abdominal wall experience less gravitational pooling of organs than in the pelvic cavity, where organs are more densely packed. These anatomical features could explain the relative rarity of adhesions at the umbilical site. However, few studies have investigated the frequency of adhesions specifically at trocar sites in laparoscopy [20,21] and even fewer have compared umbilical sites with other abdominal wall locations, indicating a need for further comparison.

Third, all SPA laparoscopic surgeries in this study were performed by a single experienced surgeon, potentially minimizing trauma to the incision site, which could reduce adhesion formation. SPA laparoscopy requires a relatively long learning curve compared to standard laparoscopy. Other studies have noted that as the learning curve for SPA procedures progresses, the incidence of wound infections significantly decreases [24]. In this study, the surgeon had performed over 3000 SPA laparoscopies [25], suggesting that surgeon skill may influence adhesion formation. Comparisons with less experienced surgeons would be useful for studying this effect.

Lastly, there is a possibility that the adhesion barrier is ineffective at the umbilical site. Guardix-SG^®^(HanmiPharmaceutical Co., Ltd., Seoul, Korea) has proven effective in various surgical specialties, including gynecology [26,27]. In this study, Guardix-SG^®^ was applied inside the pelvic cavity in both the intervention and control groups, and an additional amount was applied below the umbilicus in the intervention group. While the application of adhesion barriers in the pelvic cavity may also influence the area around the umbilicus, under the healthcare system in Korea, where the routine use of such barriers is widespread, the relative harm that could be caused to patients by not using an adhesion barrier was considered. Therefore, both groups in this study were treated with the barrier. No studies have yet assessed its application to the abdominal wall at incision sites during laparoscopic surgery. This necessitates further targeted research.

## 5. Conclusions

This research, as the first prospective randomized trial on umbilical adhesions in SPA laparoscopic surgery, provides valuable insights into adhesion prevention at the SPA incision site and confirms the safety of using adhesion barriers. Also, this study contributes to the field of personalized medicine by highlighting the need for individualized approaches to adhesion prevention in SPA laparoscopic surgery. 

In the overall patient group, the application of the adhesion barrier showed a trend towards reducing adhesions below the umbilical incision site, although this difference was not statistically significant. However, among patients without previous adhesions, the intervention group unexpectedly showed a higher incidence of adhesion formation. This finding necessitates further investigation to elucidate the underlying mechanisms and to optimize adhesion prevention strategies in laparoscopic procedures.

Potential contributing factors include the limited sample size, the anatomical features of the umbilical region, and the operating surgeon’s skill level. Nonetheless, our analysis revealed that pre-existing adhesions were a significant individual risk factor for postoperative adhesion formation, highlighting the importance of personalized risk assessment in surgical planning. Further, larger-scale studies are required to better understand the effectiveness of adhesion barriers in this context and to investigate other factors influencing adhesion formation. Future research should focus on developing personalized risk assessment tools and tailored prevention strategies to optimize outcomes for individual patients undergoing SPA laparoscopic procedures.

## Figures and Tables

**Table 1:** Baseline characteristics of participating patients.

	Total Group (*n* = 55)	Intervention Group (*n* = 25)	Control Group (*n* = 30)
Age (Years Old)	38.9 ± 8.0	38.7 ± 8.3	39.0 ± 7.9
BMI (kg/m^2^)	23.1 ± 4.4	22.1 ± 3.4	24.0 ± 5.0
Comorbidity ^1^	14 (25.5%)	6 (24%)	8 (26.7%)
ASA Score			
1	7 (12.7%)	2 (8%)	5 (16.7%)
2	48 (87.3%)	23 (92%)	25 (83.3%)
Surgical Modality			
Laparoscopy	23 (41.8%)	9 (36%)	14 (46.7%)
Robot-Assisted Surgery	32 (58.2%)	16 (64%)	16 (53.3%)
Type of Surgery			
Adnexectomy	22 (40%)	8 (32%)	14 (46.7%)
Myomectomy	16 (29.1%)	8 (32%)	8 (26.67%)
Hysterectomy	14 (25.5%)	7 (28%)	7 (23.33%)
Combined Adnexectomy and Myomectomy	2 (3.6%)	1 (4%)	1 (3.33%)
Adhesiolysis Only	1 (1.8%)	1 (4%)	0 (0%)
Endometriosis Treatment	15 (27.3%)	5 (20%)	10 (33.33%)
Operation Duration (min)	102.8 ± 44.6	114.8 ± 52.3	92.8 ± 34.9
Estimated Blood Loss (mL)	158.0 ± 207.1	192.8 ± 255.0	129.0 ± 155.4
Pre-existing Adhesions	15 (27.3%)	5 (20%)	10 (33.3%)
Blood Transfusion	1 (1.8%)	1 (4%)	0 (0%)
Hemoglobin Change (g/dL)	1.57 ± 1.02	1.62 ± 1.08	1.53 ± 0.98
Hospital Stay (Days)	3.6 ± 0.6	3.7 ± 0.6	3.5 ± 0.6

^1^ Comorbidity includes hypertension, diabetes, hyperlipidemia, etc.

**Table 2:** Postoperative complications of participating patients.

	Total Group (*n* = 55)	Intervention Group (*n* = 25)	Control Group (*n* = 30)	*p*-Value
Visceral movement during				
Tidal Respiration (cm)	1.39 ± 0.45	1.40 ± 0.44	1.38 ± 0.46	0.891
Maximum Respiration (cm)	2.99 ± 1.02	3.00 ± 1.03	2.97 ± 1.03	0.925
Postoperative Adhesions	6 (10.9%)	2 (8%)	4 (13.3%)	0.678
Wound Infection	3 (5.5%)	1 (4%)	2 (6.7%)	1
Wound Dehiscence				
Superficial dehiscence	1 (1.8%)	0 (0%)	1 (3.3%)	1
Deep dehiscence	0 (0%)	0 (0%)	0 (0%)	
Incisional Hernia	0 (0%)	0 (0%)	0 (0%)	

**Table 3:** Postoperative complications of patients who did not have pre-existing adhesions.

	Total Group (*n* = 40)	Intervention Group (*n* = 20)	Control Group (*n* = 20)	*p*-Value
Visceral movement during				
Tidal Volume (cm)	1.38 ± 0.44	1.32 ± 1.08	1.43 ± 0.48	0.440
Maximum Respiration (cm)	2.92 ± 0.96	2.67 ± 0.75	3.12 ± 1.08	0.134
Postoperative Adhesions	2 (5%)	2 (10%)	0 (0%)	0.487
Wound Infection	2 (5%)	1 (5%)	1 (5%)	1
Wound Dehiscence	0 (0%)	0 (0%)	0 (0%)	

## Data Availability

The original contributions presented in this study are included in the article. Further inquiries can be directed to the corresponding author.

## Supplementary Materials

The following supporting information can be downloaded at: https://www.mdpi.com/article/10.3390/jpm15020068/s1, Video S1: Visceral sliding technique via trans-abdominal ultrasound.
